# Supplementary material for: Therapeutic vulnerability to PARP1,2 inhibition in RB1-mutant osteosarcoma
Source: Nat Commun. 2021 Dec 3;12:7064. doi: 10.1038/s41467-021-27291-8 (PMC8642453; doi:10.1038/s41467-021-27291-8)
Supplement: Supplementary file 1 — Supplementary Information [file 41467_2021_27291_MOESM1_ESM.pdf]

SUPPLEMENT: Therapeutic vulnerability to PARP1,2 inhibition in *RB1*-mutant osteosarcoma

Georgia Zoumpoulidou, Carlos Alvarez-Mendoza, Caterina Mancusi, Ritika-Mahmuda Ahmed, Milly Denman, Christopher D Steele, Maxime Tarabichi, Errin Roy, Lauren R Davies, Jiten Manji, Camilla Cristalli, Katia Scotlandi, Nischalan Pillay, Sandra J Strauss, Sibylle Mittnacht*

***corresponding author**

SUPPLEMENTARY FIGURES AND LEGENDS
SUPPLEMENTARY TABLES AND LEGENDS
SUPPLEMENTARY MATERIAL AND METHODS INFORMATION
SUPPLEMENTARY REFERENCES

SUPPLEMENTARY FIGURES AND FIGURE LEGENDS

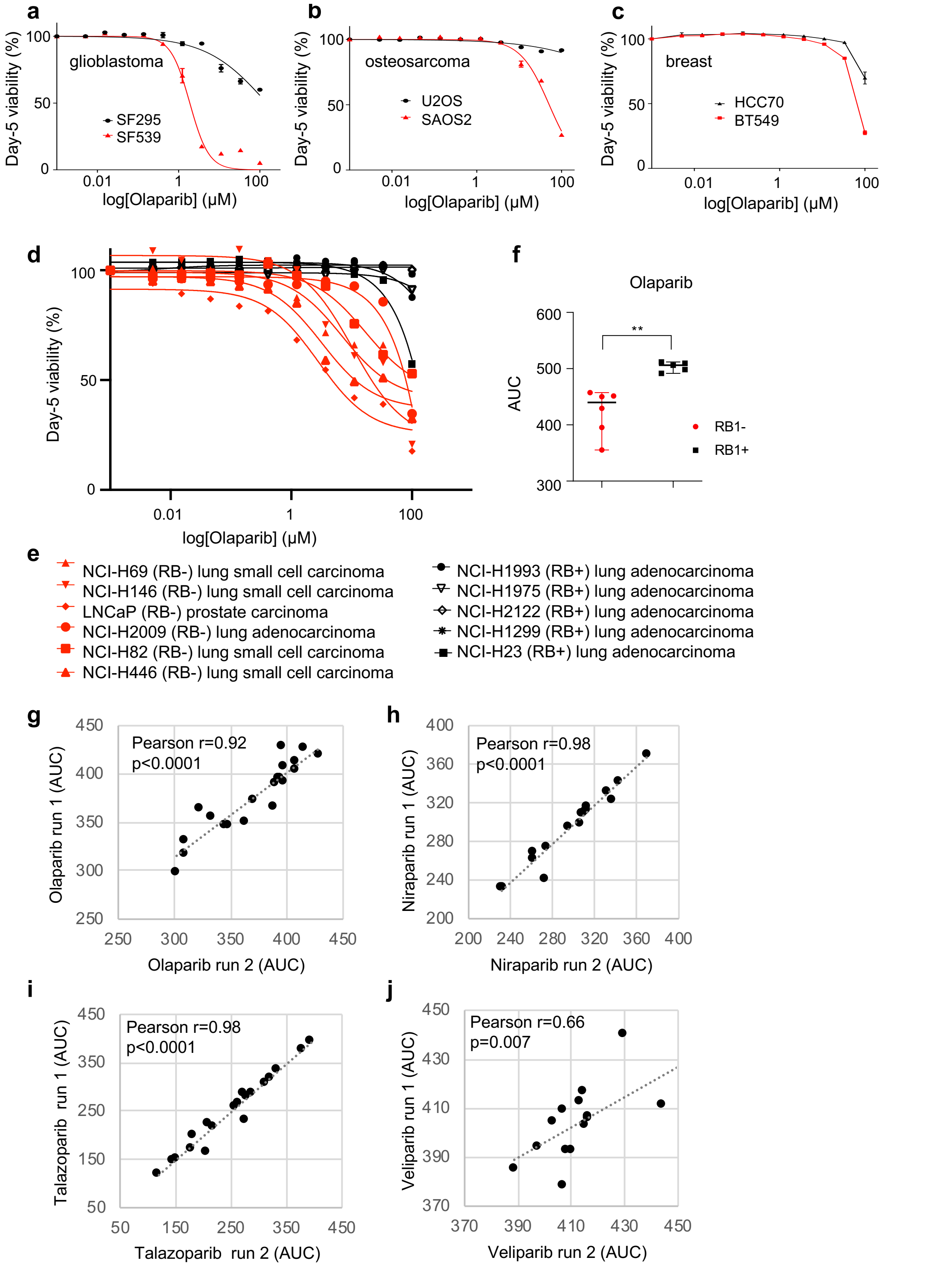

Supplementary Figure 1: Differential PARP inhibitor (PARPi) sensitivities in RB1-defective and RB1-normal tumour cell lines. Cell seeded in 96-well plates were treated with PARPi at concentrations indicated. Cell viability was determined 5 days after inhibitor addition using resazurin reduction.

a-c) Histiotype-matched cancer cell lines with differing *RB1* mutation status, assessed for sensitivity to PARPi olaparib. Day-5 viability concentration-response curves depicting the decrease in resazurin signal in PARPi-treated samples relative to the DMSO-treated controls for **a)** glioblastoma, **b)** osteosarcoma, **c)** breast. Results shown depict data for one of n = 2 biologically independent datasets. Data points depict the mean of three parallel replicates. RB1-defective (red), RB1-normal status (black).
d-f) Day-5 viability assessment across a poly-cancer cell line panel, with **d)** concentration-response curve, **e)** Symbols, names and histiotype of cell lines used and f**)** Scatter plot for AUC values deduced from dose-response curves. Error bars in **f** indicate median and 95% CI Red, RB1-defective and black, RB1-normal status, calculated using a two-tailed Mann-Whitney test, p (f) =0.0043**. Data **(d-f)** depict one of n = 2 biologically independent datasets. Data points represent the mean of three parallel replicates.
g-j) Pearson product moment correlation measuring the strength of a linear association between AUC values deduced from day-5 viability concentration-response curves shown in Figure 1. Graphs compare n = 2 biologically independent datasets with data values representing the mean of three parallel replicates. Treatment entailed **g)** olaparib, **h)** niraparib, **i)** talazoparib and **j)** veliparib. Pearson's correlation coefficient r, and p-values relating to r, are shown for two-tailed tests. Data relate to Figure 1.
Source data are provided as a Source Data file.

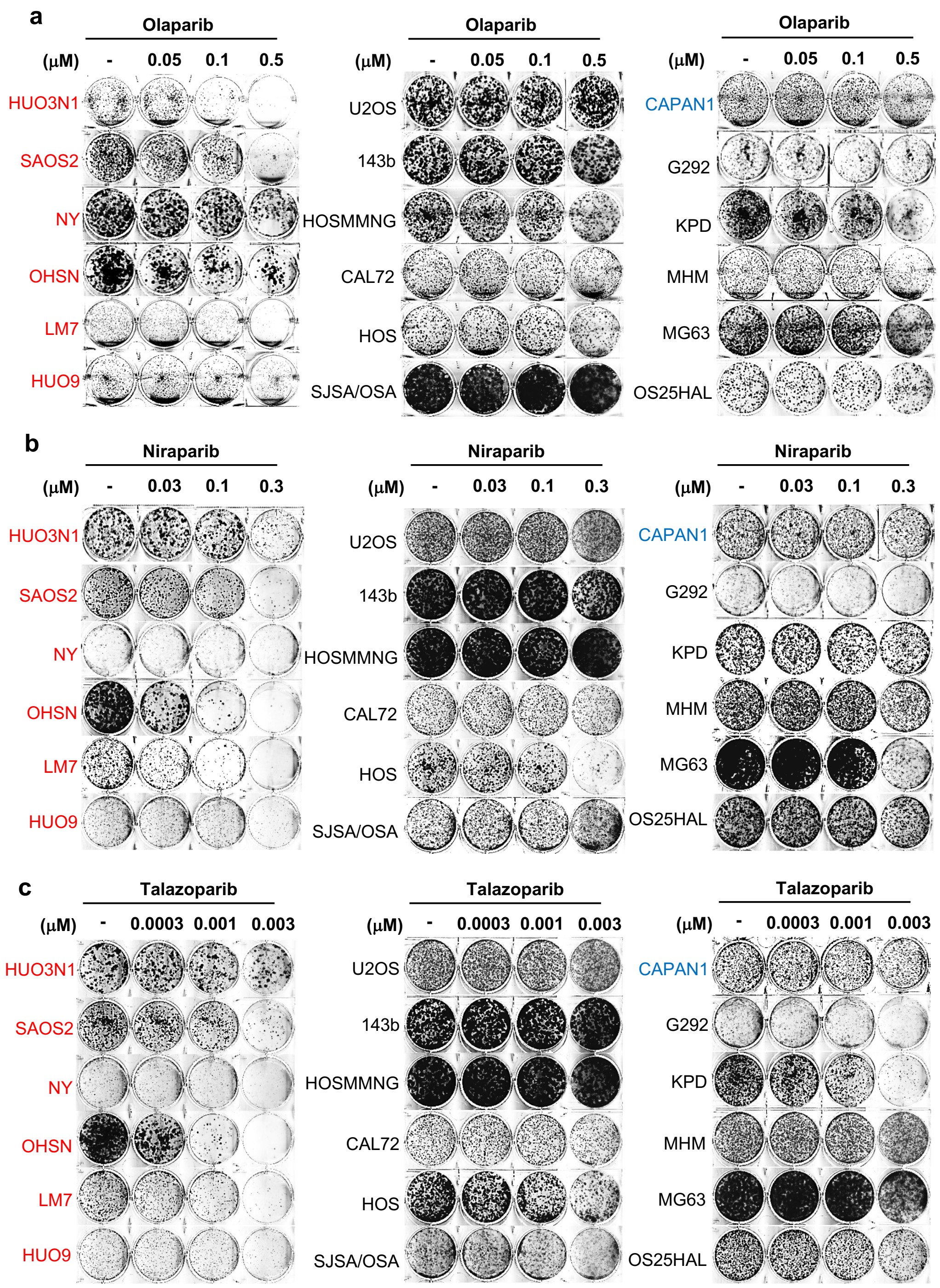

Supplementary Figure 2: Effect of PARP inhibition assessed using clonogenic survival assays and depicting raw plate images. Osteosarcoma tumour cell lines differencing in RB1-status were seeded into 6-well plates in the presence of vehicle (DMSO) or increasing concentrations of three PARP inhibitors. Cells were treated, fixed after 12-14 days and stained using crystal violet dye. Representative raw plate images of plates stained with crystal violet after treatment with **a)** olaparib, **b)** niraparib and **c)** talazoparib. RB1-defective (red), RB1-normal status (black). Data related to Figure 2.

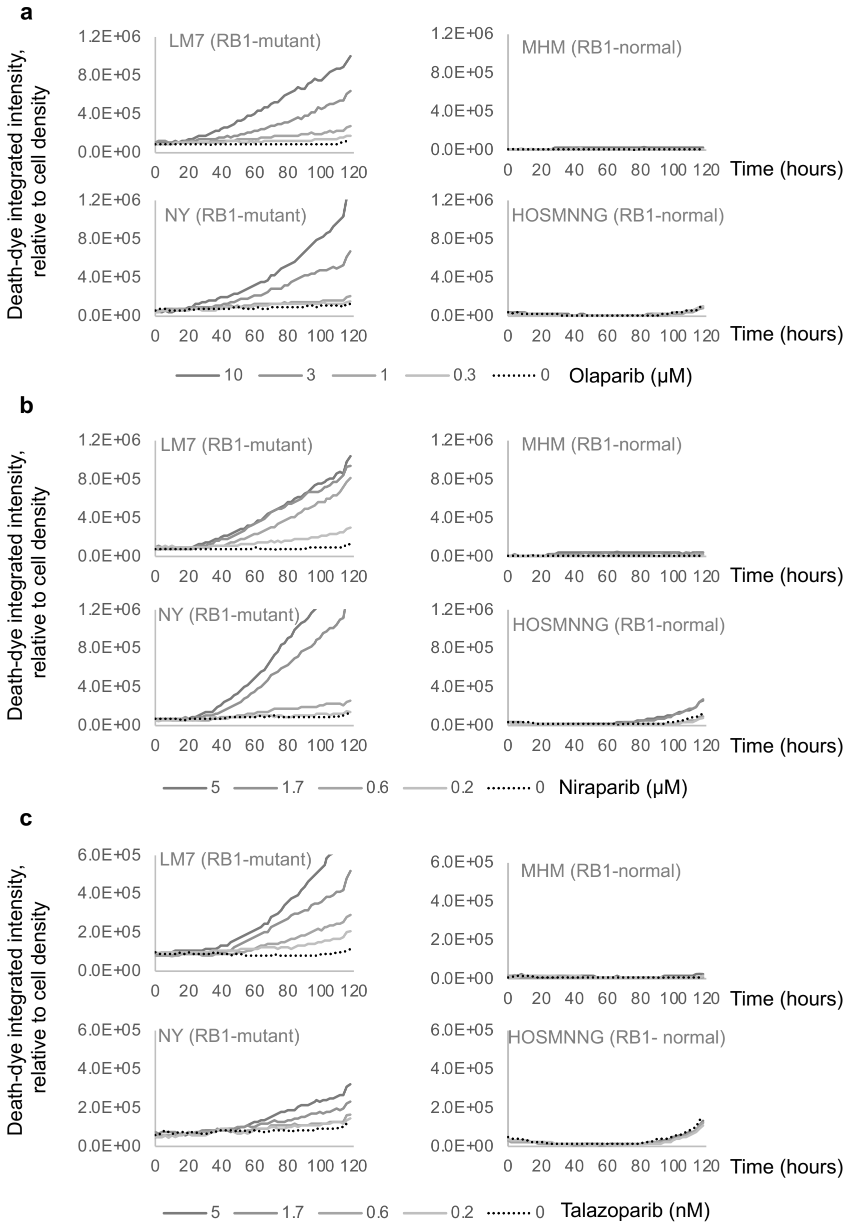

Supplementary Figure 3: Cellular effects of PARPi treatment. RB1-defective (LM7 and NY) and RB1-normal (MHM and HOSMNNG) osteosarcoma cell lines were treated in parallel with PARP inhibitors olaparib, niraparib and talazoparib, then subjected to time-lapse microscopy in the presence of SYTOX^{TM} death-dye. Images were taken every two hours, recording phase contrast and death-dye fluorescence. Inhibitors were used at an equipotent concentration range deduced using clonogenic survival assays.

a-c) Graphs depicting death-dye incorporation over time relative to cell density with **a)** cells treated with olaparib, **b)** cells treated with niraparib, **c)** cells treated with talazoparib. Data represent one of n = 2 biologically independent datasets.

Source data are provided as a Source Data file.

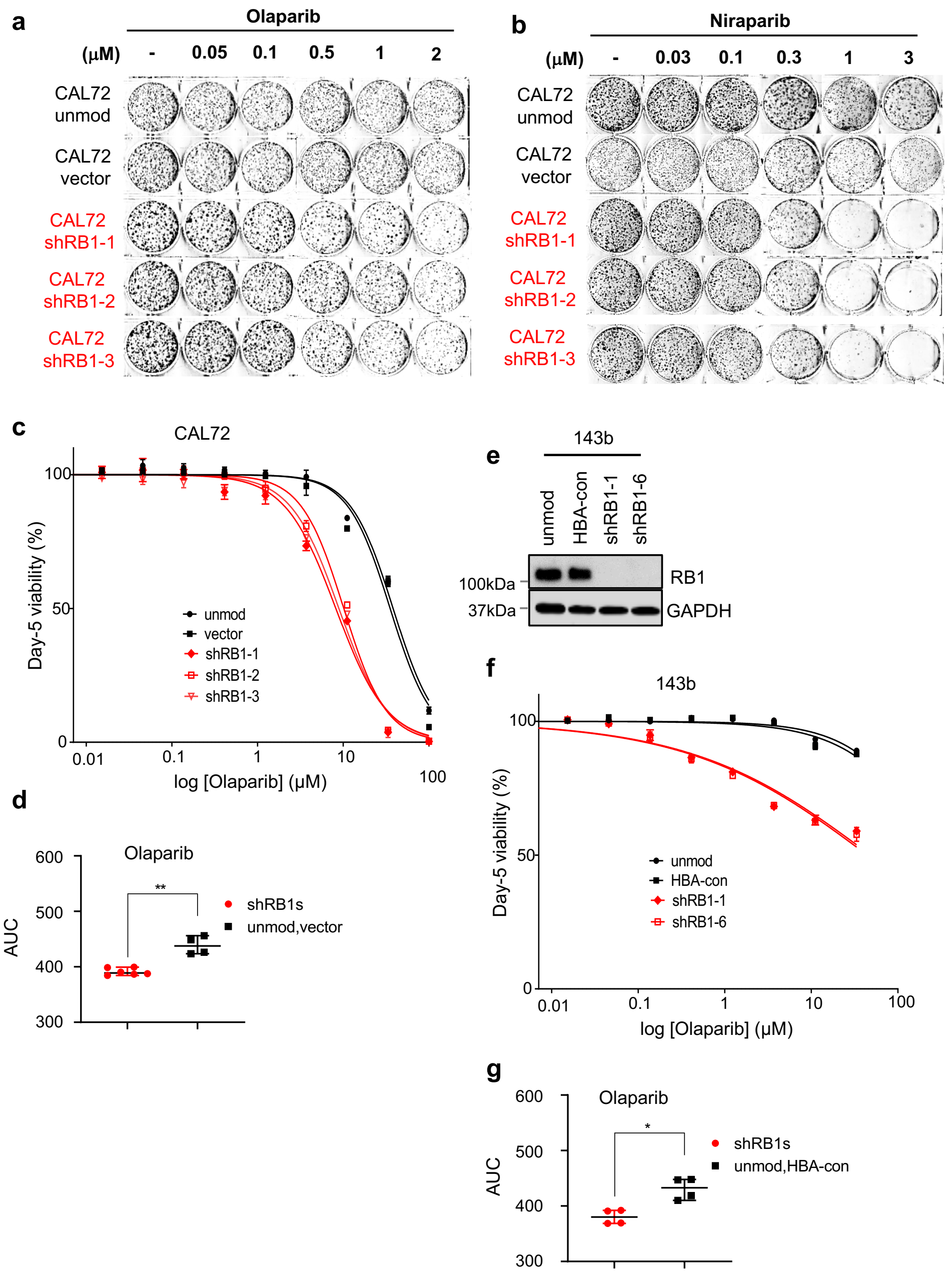

Supplementary Figure 4: Effect of PARP inhibition in RB1-normal osteosarcoma CAL72 and 143b following RB1 depletion.

CAL72 and 143b cells were transduced with lentivirus vector encoding *RB1*-targeting shRNAs shRB1-1, shRB1-2, shRB1-3, shRB1-6, empty vector backbone (vector), or irrelevant control hemoglobin (HBA)-targeting shRNA or left unmodified (unmod).

a-b) Representative raw plate images illustrating clonogenic survival in RB1-normal osteosarcoma CAL72 cells with shRNA-mediated RB1 ablation. Cells were treated with **a)** olaparib, **b)** niraparib. Data related to Figure 4.

c-d, f-g) Day-5 viability assessment. Cell seeded in 96-well plates were treated with PARPi olaparib at concentrations as indicated. Cell viability was determined 5 days following inhibitor addition using resazurin-reduction. Concentration-response curves for **c)** CAL72 or **f)** 143b.

Graphs depict results for one of n = 2 biologically independent datasets, data points represent the mean of three parallel samples relative to the DMSO-treated controls. Scatter plots depicting AUC values deduced from dose-response curves for **d)** CAL72 or **g)** 143b summarising results for n = 2 biologically independent datasets. Bars depict median and 95% confidence interval (CI), p (d) =0.0095 and p (g) =0.0286 using a two-tailed Mann-Whitney test, *p<0.05, **p<0.01. **e)** Immunoblot analysis documenting RB1 expression on 143b cells. GAPDH was used as a loading control.
Source data are provided as a Source Data file.

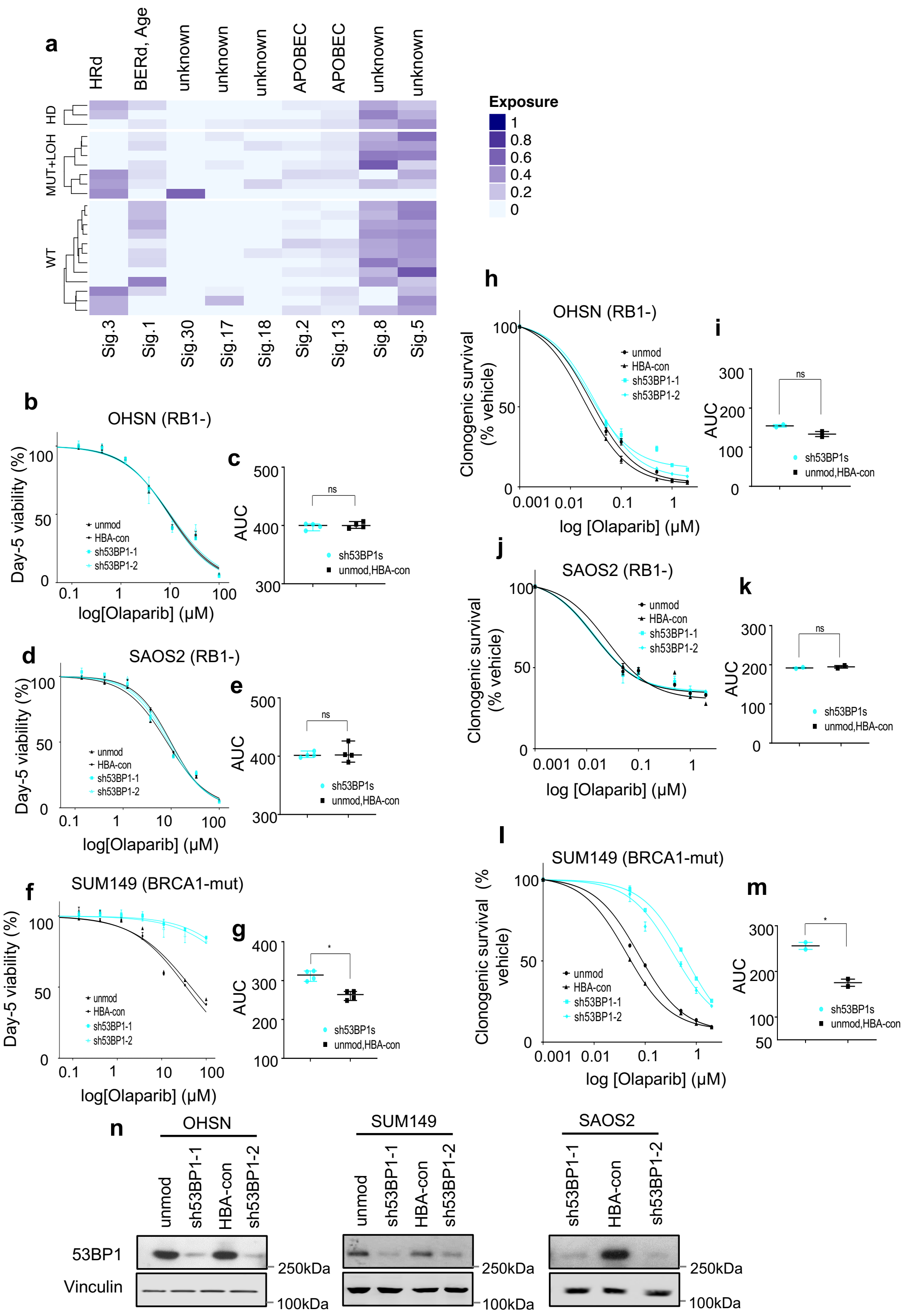

Supplementary Figure 5: Mutational signature analysis for published osteosarcoma whole-exome data and effect of HR de-repression through 53BP1-ablation in RB1-defective osteosarcoma cells.

a) Mutational data from 37 previously published whole-genome sequenced osteosarcomas (4) was downloaded. Cosmic V2 signature classification with associated etiology prediction is shown. Etiology terms; Homologous recombination defect (HRd), Base excision repair defect (BERd), apolipoprotein B mRNA editing enzyme (APOBEC). Homozygous deletions, CN [1] and

loss-of-heterozygosity events, CN = {>0,0} and mutational data were called at *RB1* to determine *RB1* mutation status.

b-n) PARPi sensitivity following 53BP1 depletion. RB1-defective osteosarcoma OHSN and SAOS2 and *BRCA1*-mutant breast cancer SUM149 cells were transduced with lentivirus vector encoding 53BP1-targeting shRNAs sh53BP1-1 and sh53BP1-2, an irrelevant control hemoglobin (HBA)-targeting shRNA or left unmodified (unmod). ^{ns}p>0.05, *p<0.05, calculated using a two-tailed Mann-Whitney test. **(b-g**) Day-5 viability assessments using **b-c)** OHSN, **d-e)** SAOS2, and **f-g)** SUM149 treated with Olaparib, p (g) =0.0286 *. Shown are concentration-response curves **(b, d, f)** for one of n = 2 biologically independent datasets, data points representing the mean of three parallel replicate samples, and scatter plots **(c, e, g)** summarizing AUC values for n = 2 biologically independent datasets. Bars depict median and 95% confidence interval (CI). **h-m)** Clonogenic survival analysis assessing PARPi sensitivity following 53BP1 depletion for **h-i)** OHSN, **j-k)** SAOS2 and **l-m)** SUM149 cells treated with Olaparib, p (m) =0.0181 *. Shown are concentration-effect curves and AUC scatter plots. Data reflect the mean +/-SD of parallel duplicate wells for one dataset. Bars depict median and 95% CI, **n)** Immunoblot analysis documenting 53BP1 expression. Vinculin was used as a loading control.

Source data are provided as a Source Data file.

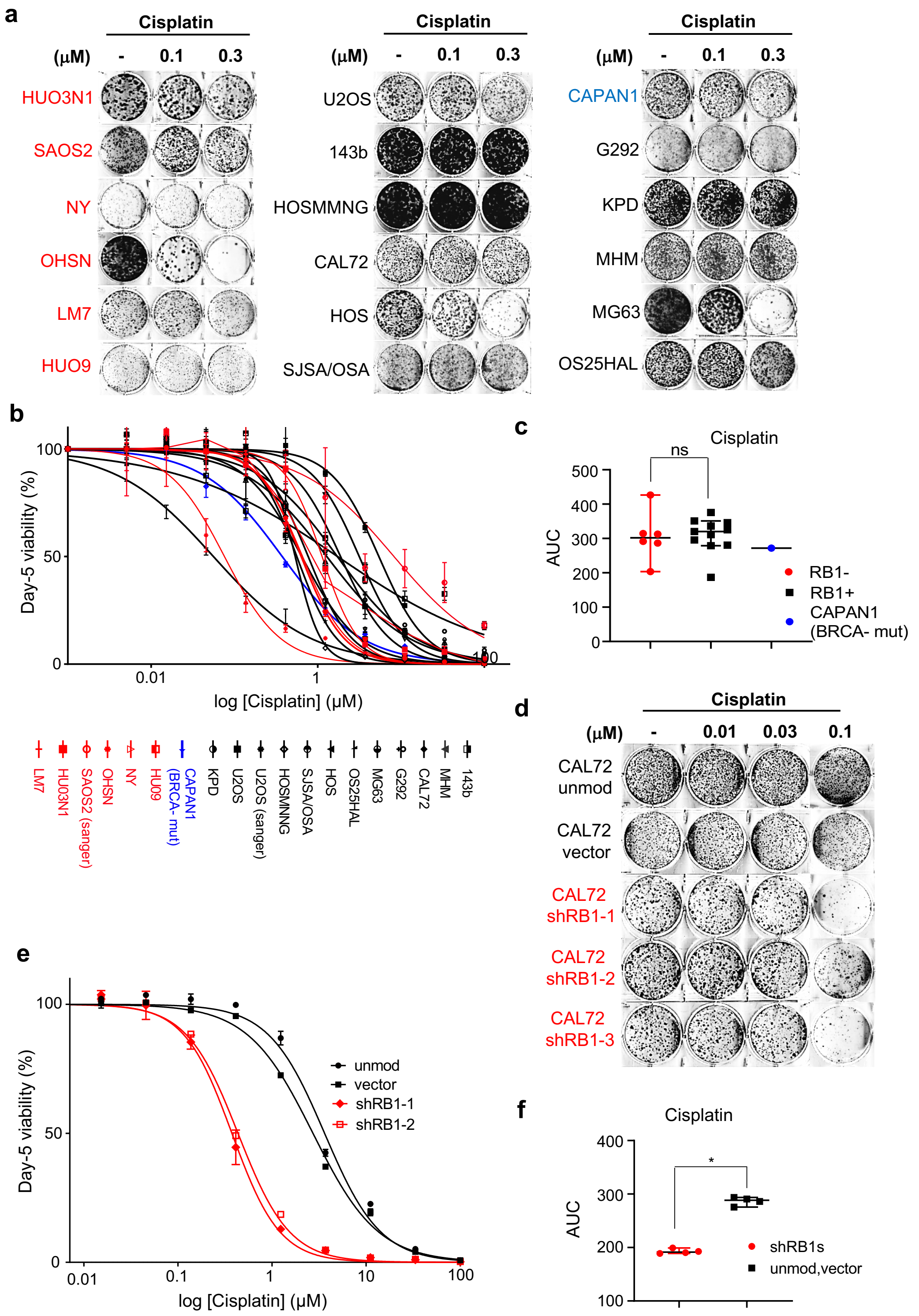

Supplementary Figure 6: Platinum sensitivity in RB1-defective osteosarcoma.

a-c) Platinum response in RB1-defective (red) or RB1-normal (black) osteosarcoma lines. **a)** Representative raw plate images illustrating clonogenic survival following culture in the presence of increasing concentrations of cisplatin. Data related to Figure 6a-c. **b-c)** Day-5 viability assessment. Graphs depict **b)** Concentration-response curves, reflecting mean +/-SD of parallel triplicate wells for one of n = 2 biologically independent datasets and **c)** AUC value

comparison calculated using two-tailed Mann-Whitney test, p (c) =0.66 ^{ns}. Data summarise outcome from n = 2 biologically independent datasets. Bars depict median (±95%CI).

d-f) Platinum response in RB1-normal osteosarcoma CAL72 cells with shRNA-mediated RB1 ablation. **d)** Representative raw plate images illustrating clonogenic survival following culture in the presence of increasing concentrations of cisplatin. **e-f)** Day-5 viability in RB1-normal osteosarcoma CAL72 cells with shRNA-mediated *RB1* ablation, determined using resazurin-reduction. Graphs depict, **e)** Concentration-response curves, reflecting mean +/-SD of parallel triplicate wells for one representative experiment, and **f)** AUC value comparison calculated using two-tailed Mann-Whitney test, p (f) =0.029 *. Data summarise outcome from n = 2 biologically independent datasets. Bars depict median (±95%CI), Data relate to Figure 6. Source data are provided as a Source Data file.

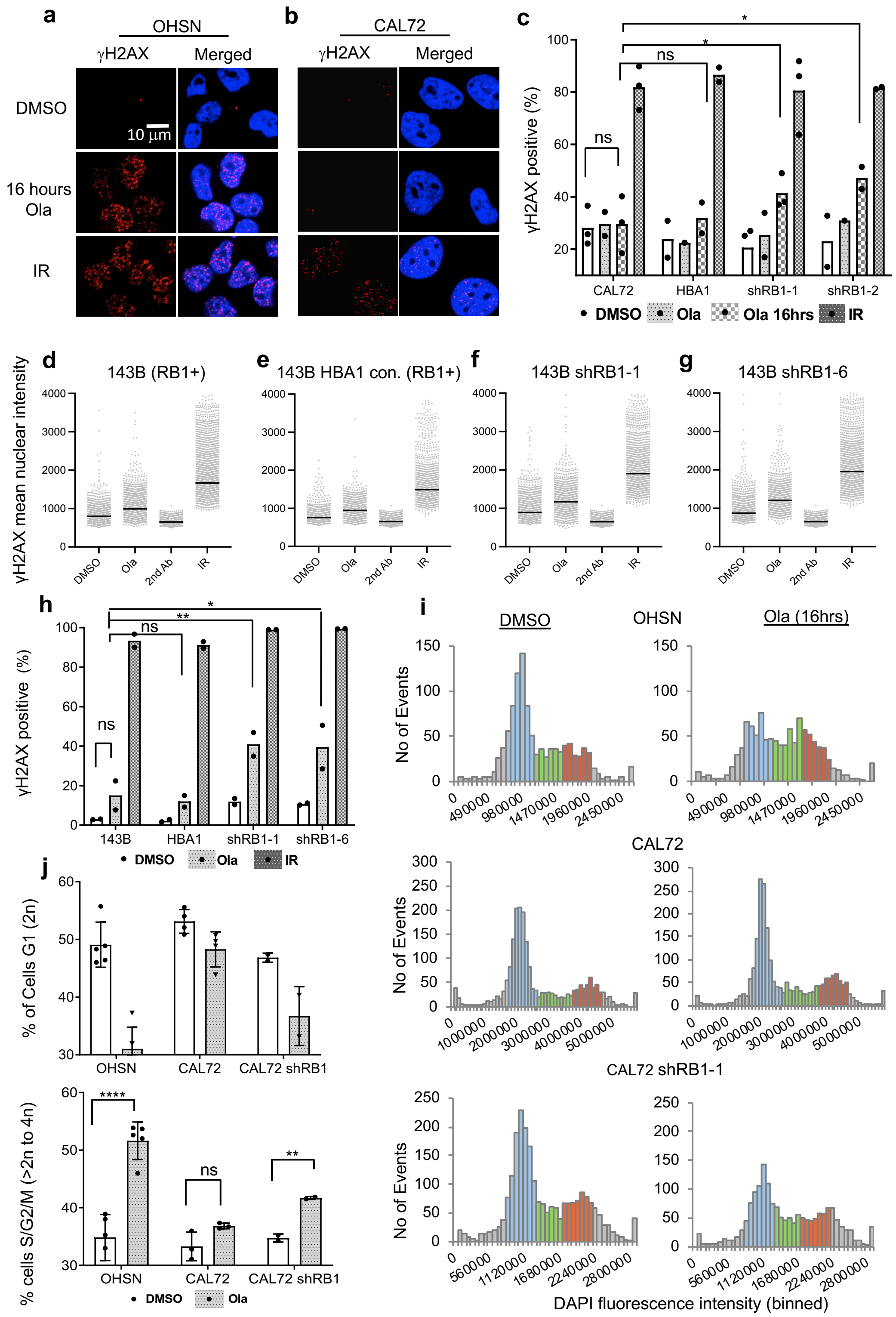

Supplementary Figure 7: Effect of PARP inhibition on DNA damage response in cells with different RB1 status. a, b) Representative images for anti-γH2AX- immunostaining related to Figure 7a. **a)** RB1-defective OHSN and **b)** RB1-normal CAL72. Cells were either irradiated (2Gy) and fixed 1 hour later or treated with 3 µM of olaparib or vehicle (DMSO) for

16 hrs. Cells were immunostained with anti-γH2AX. Cell nuclei were counterstained with DAPI. Scale bar, 10 μm. Data relate to Figure 7.

c) Bar graph relating to Figure 7b, depicting the mean percentage of cells with anti-γH2AX immunofluorescence above that observed in vehicle treated cells (% γH2AX-positive) for n = 3 (CAL72 and CAL72 shRB1-1) or n = 2 (CAL72 shRB1-2, and HBA1) biologically independent datasets, respectively. Cell lines as indicated were treated as for Figure 7b, using 3 μM olaparib for the time indicated, vehicle (DMSO) for 16 hrs, or 2.5 Gy of IR with 1-hour recovery. p (Ola 16hrs, CAL72 DMSO) = 0.9173, p (Ola 16hrs, CAL72-HBA1) = 0.3946, p (Ola 16hrs, CAL72-shRB1-1) = 0.0109, p (Ola 16hrs, CAL72-shRB1-2) = 0.0302, calculated using a two-sided, one-way ANOVA test, $^{ns}p>0.05$, $^{*}p<0.05$, $^{**}p<0.01$. Data relate to Figure 7.

d-h) DDSB repair signalling response assessed using anti-γH2AX in RB1-normal osteosarcoma 143b and 143b transduced with lentivirus vector encoding shRNA targeting *RB1* or irrelevant control hemoglobin (HBA)-targeting shRNA. Scatter plots depicting raw data distributions with median intensity for one of n = 2 biologically independent datasets in **d-g**, and bar graph depicting the mean percentage of cells with anti-γH2AX immunofluorescence above that observed in vehicle-treated cells (+/- SD) (% γH2AX-positive) for n = 2 biologically independent datasets in **h**. Cells were treated as indicated. p (Ola 16hrs, 143b DMSO-Ola) =0.1253, p (Ola 16hrs, 143b-HBA1) =0.7007, p (Ola 16hrs, 143b-shRB1-1) =0.0086, p (Ola 16hrs, 143b-shRB1-2) =0.01, calculated using a two-sided, one-way ANOVA test. $^{ns}p>0.05$, $^{*}p<0.05$, $^{**}p<0.01$.

i, j) Cell cycle response analysis using quantitative fluorescence microscopy-derived single-cell resolved DAPI intensity. Cells, as indicated, were treated for 16 hours with vehicle or 3 μM olaparib for 16 hours. Raw sum DAPI intensity profiles for RB1-defective OHSN (representative for n = 5 biologically independent datasets), unmodified RB1-normal CAL72 (representative for n = 4 biologically independent datasets) and *RB1*-depleted CAL72 (representative for n = 2 biologically independent datasets) in **i.** The colouration depicts gating strategy used for each cell cycle phase, where blue = 2n (G1), green >2n to <4n (S) and Red = 4n (G2). Bar graph summarising data for across datasets in **j**. Bars reflect the mean percentage (+/- SD) of cells in G1 or S/G2/M phase of the cell cycle for each of the cell lines across all datasets. p (OHSN DMSO-Ola 16hrs S/G2/M) <0.0001, p (CAL72 DMSO-Ola 16hrs S/G2/M) =0.3946, p (CAL72-shRB1 DMSO-Ola 16hrs S/G2/M) =0.0057, calculated using an unpaired, two-tailed Student's t-test. $^{ns}p>0.05$, $^{**}p<0.01$, $^{****}p<0.0001$

Source data are provided as a Source Data file.

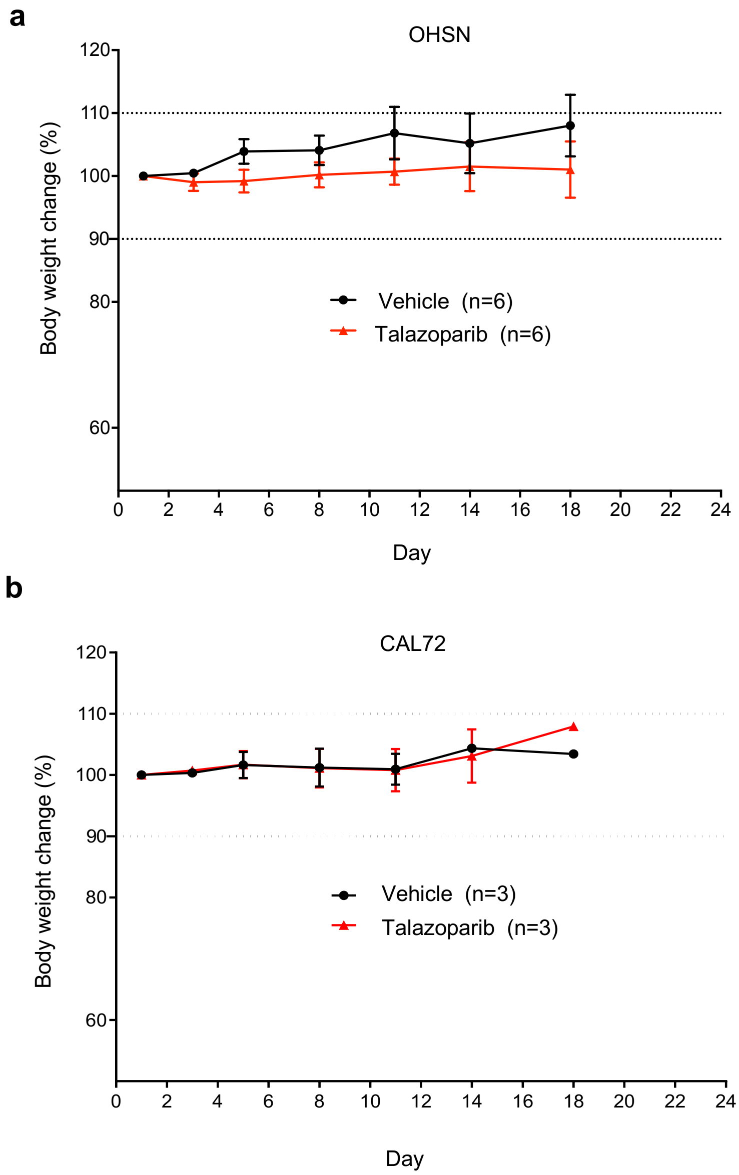

Supplementary Figure 8: Effect of PARPi treatment on OHSN and CAL72 xenograft-bearing mice. a, b) Bodyweight over time in mice treated with 0.33 mg/kg talazoparib or vehicle p. o. (mean ±SD, n = 6 or n = 3 per group). Data represent % change in weight relative to treatment start, the dotted lines mark 10% differential boundaries of acceptable weight fluctuation. Data related to Figure 9.

Source data are provided as a Source Data file.

SUPPLEMENTARY TABLES

Cell lines	Olaparib	Cell lines	Niraparib	Cell lines	Talazoparib	Cell lines	Cisplatin
	IC50 (µM)		IC50 (µM)		IC50 (nM)		IC50 (µM)
(RB1-)		(RB1-)		(RB1-)		(RB1-)	
HUO3N1	0.04± 0.02	LM7	0.02	OHSN	0.3± 0.1	OHSN	0.03± 0.01
OHSN	0.05 ± 0.03	OHSN	0.02± 0.01	LM7	0.43	HUO3N1	0.34± 0.01
LM7	0.11 ± 0.01	SAOS2	0.20± 0.01	SAOS2	2.00± 0.0	NY	0.46
NY	0.13 ± 0.01	NY	0.48	NY	2.00	LM7	0.52
SAOS2	0.52 ± 0.53	HUO3N1	0.51± 0.04	HUO9	2.00	SAOS2	0.57± 0.01
HUO9	0.56 ± 0.03	HUO9	0.55	HUO3N1	3.5± 0.6	HUO9	0.79
Median	0.12	Median	0.34	Median	2.00	Median	0.49
(RB1+)		(RB1+)		(RB1+)		(RB1+)	
G292	0.23 ± 0.12	MG63	0.18	KPD	1.00	HOS	0.06
KPD	0.40 ± 0.02	HOS	0.21	HOS	3.00	MG63	0.19
MG63	1.30 ± 0.01	KPD	0.33	MG63	4.00	HOSMNNG	0.34± 0.02
OS25HAL	1.32 ± 0.01	143b	0.55± 0.06	OS25HAL	7.00	U2OS	0.39
SJSA/OSA	1.33 ± 0.04	OS25HAL	0.86	HOSMNNG	11.5± 3.5	OS25HAL	0.54
HOS	1.83± 0.0	CAL72	1.36± 0.05	G292	16.00	143b	0.63± 0.03
HOSMNNG	3.03 ± 0.03	MHM	1.46	SJSA/OSA	18.00± 1.4	KPD	0.75
CAL72	§± 0.0	HOSMNNG	1.52± 0.06	143b	22.00± 1.3	G292	§
MHM	§± 0.0	G292	1.72	MHM	55.00	CAL72	§± 0.0
U2OS	§± 0.0	U2OS	1.74	U2OS	77.00	MHM	§
143B	§± 0.0	SJSA/OSA	1.78± 0.03	CAL72	§± 0.0	SJSA/OSA	§± 0.0
Median	n.d	Median	n.d	Median	n.d.	Median	n.d
(BRCA-)		(BRCA-)		(BRCA-)		(BRCA-)	
CAPAN1	1.41 ± 0.29	CAPAN1	0.21	CAPAN1	3.00	CAPAN1	0.19

Supplementary Table 1: Inhibitory constant 50 (IC50) values based on inhibition of clonogenic activity +/-SD for two or more datasets. Cell lines grouped for (RB-) RB1-defective, (RB+) RB1-normal, (BRCA) *BRCA2*-mutated. Median denotes median IC50 values calculated for the respective groups. n.d. = not determined. § denotes cases where an IC50 could not be extrapolated from the concentration-response data observed.

	Olaparib	Niraparib	Cisplatin
Cell line	IC50 (µM)	IC50 (µM)	IC50 (µM)
CAL72 unmod	§ ± 0.0	2.177 ± 0.25	§ ± 0.0
CAL72 vector	§ ± 0.0	1.376 ± 0.12	§ ± 0.0
CAL72 shRB1-1	0.408 ± 0.28	0.249 ± 0.03	0.239 ± 0.15
CAL72 shRB1-2	0.563 ± 0.23	0.175 ± 0.01	0.202 ± 0.01
CAL72 shRB1-3	0.493 ± 0.0	0.377 ± 0.08	0.088 ± 0.01

Supplementary Table 2: Inhibitory constant 50 (IC50) values in CAL72 RB1-normal osteosarcoma with and with shRNA-mediated *RB1* ablation. Data are based on inhibition

of clonogenic activity for n = 2 datasets. § denotes situations where IC50 values could not be extrapolated from the concentration range assessed.

SUPPLEMENTARY METHOD AND MATERIAL INFORMATION

Cell lines, chemicals and antibodies. All cell lines were maintained at 37°C in a humidified incubator with 5% CO_2. STR profiling using the GenePrint 10 system (Promega) was used to confirm identity for established osteosarcoma lines. cc hosts [2] Patient informed consent was obtained for the establishment of these PDX models. Absence of mycoplasma contamination was confirmed using the Mycoalert Mycoplasma Detection Kit, Lonza. Lines were authenticated against the profile of the original PDX by DNA Fingerprinting (POWERPLEX ESX 17 Fast System, Promega) before use. Cisplatin and PARP inhibitors olaparib, niraparib, veliparib and talazoparib were purchased from Selleck Chemicals, dissolved in DMSO as a 10mM stock solution and stored at -80°C. Thymidine (Sigma), was dissolved in phosphate-buffered saline (PBS) and used at a final concentration of 2 mM. For immunoblotting the following antibodies were used: anti-RB1 (4H1), anti-phospho-CHK1 (Ser345) (133D3), anti-phospho-CHK2 (Thr68) (C13C1), anti-CHK1 (2G1D5), anti-CHK2 (1C12) were purchased from Cell Signalling Technology, anti-GAPDH (6C5) and anti-53BP1 (EPR2172) were obtained from Abcam, anti-Vinculin (hVIN-1) was obtained from Sigma. For immunofluorescence analysis, anti-RAD51 (ab176458) (Abcam) and anti-phosphorylated histone-H2AX (Ser139) (JBW301) (Merck Millipore) were used.

Clonogenic survival assays. Assays were performed as previously described [3]. Cells were seeded in 6 -well plates at a concentration of 1000-2000 cells per well and on the following day treated with vehicle or inhibitors. Two independent wells were used per conditions. After 10 to 14 days, plates were washed with Phosphate Buffered Saline (PBS), cell layers fixed with 4% formaldehyde for 10 minutes and stained for a minimum of 20 minutes using 0.025% f. c. crystal violet dye in PBS. Excess dye was rinsed off using repeat immersion in tap water. Plates were air-dried and images were captured using a desktop scanner. To quantify colony formation proficiency bound crystal violet dye was extracted using 30% v/v acetic acid, 30% v/v DMSO and 0.1% SDS and the optical absorbance was determined spectrophotometrically at 570 nm using a multi-functional microplate reader. Clonogenic activity for inhibitor-treated cells is expressed relative to that of vehicle-treated wells. Concentration-response curves were plotted using a three-parameter regression fit in GraphPad Prism 8.

Immunofluorescence. Cells were seeded on glass coverslips placed on 6-well plates. 24 hours after plating cells were treated with inhibitors or ionising radiation. For analysis, cells were fixed in 4% paraformaldehyde in PBS for 10 minutes, washed twice with TBS, permeabilised with 0.2% Triton X-100 in PBS for 10 minutes, and incubated in 2% BSA in PBS for 20 minutes. Cells were then incubated at room temperature for 2 hours with primary antibodies diluted according to the manufacturer's recommendation in 2% BSA in TBS-T. Cells were washed in three changes of PBS followed by incubation with ALEXA Fluor 488 or 568 coupled secondary antibodies, diluted in 2% BSA in TBS-T for 1 hour. Cells were washed in three changes of PBS and mounted using VECTASHIELD^{TM} (Vector laboratories) containing the DNA intercalating dye DAPI. For datasets where DNA content was derived using DAPI incorporation, cells were incubated with 0.5 µg/ml DAPI in PBS for 20 minutes, Cells thereafter were washed in three changes of PBS and mounted using VECTASHIELD without DAPI.
RAD51 staining was visualised using an LSM 510 Zeiss confocal laser-scanning microscope using the 63 X 1.4 NA oil objective. Z stacks were taken of cells representative and Maximum Intensity projections generated. For quantitative analysis, ≥ 100 cells from each condition were chosen at random and nuclear foci were counted manually to determine the percent positive for RAD51. Singe-cell-resolved γH2AX mean intensity and DAPI sum intensity was deduced following collection of micrographs by a Zeiss Axio imager A1 microscope using Imaris Cell Imaging software (Oxford Instruments). A mask was created, which defined cell nuclei using the DAPI channel fluorescence. From this, single-cell resolved, quantitative data was deduced for each stain and exported to Microsoft Excel for analysis. At least 100 nuclei were analysed per experiment and condition. Gates to derive the % γH2AX-positive cells were set based on the distribution of fluorescence in vehicle-treated cells, essentially as described in [4] [5]. Fluorescence intensity integrated over the measured area (sum intensity) was used as the quantity/ cell for DAPI staining, mean fluorescence was used as a quantity/ cell for γH2AX, based on consideration described in [5].

Immunoblotting. Whole-cell protein extracts were prepared by lysis of cells into SDS buffer (0.1% SDS, 50 mM TRIS-HCL, pH 6.8. containing EDTA free protease inhibitors (Pierce) and phosphatase inhibitors Na-vanadate at 2mM, β-glycerophosphate at 10 mM and NaF at 10mM final concentration). Lysis buffer was made up fresh and heated to 80°C prior to lysate preparation. Samples were diluted to contain equal protein concentration prior to analysis. Protein concentrations in samples were measured by BCA protein assay (Pierce). Samples were separated on SDS polyacrylamide gels and transferred to an Immobilon-FL membrane (Millipore), before incubation with primary antibodies overnight at 4 °C. Membranes were washed with TBST (25 mM Tris, 140 mM NaCl, 0.1% Tween-20, pH 7.5) and probed with

IRDye 680- or IRDye 800CW-conjugated (LI-COR), or HRP-conjugated secondary antibodies for 1 hour at room temperature. The imaging and quantification of signals were carried out using an Odyssey CLx infrared imaging system or chemiluminescent detection (Pierce).

Time-resolved cell death analysis. Cells were seeded the day before the experiment at 10000-20000 cells per well of a 96 well plate or 40000 cells per well of a 12 well plate. The following day cells were treated with inhibitors at the indicated doses in the presence of 1μM SYTOX™ Green (Thermofisher) or 0.1μM Propidium Iodine (Sigma). Cells were imaged in real-time for the incorporation in 2 hours intervals using an IncuCyte^{ZOOM} system, (essenbiosciences), as described before in Zhang C. et.al, 2019.

Day-5 viability assessment using resazurin. Resazurin reduction assays were used to quantify the effect on inhibitor treatment cell survival and proliferation. Briefly, cells were seeded (1000-4000 cells/well) at densities optimised for each cell line in parallel triplicate wells on clear, flat-bottom 96-well plates (Corning). The following day, cells were treated with increasing concentrations of inhibitors at the indicated doses for 5 days added to wells as a 10-fold concentrated working stock, generated in cell culture media. Assessments were run in triplicate. Five days following drug addition, resazurin dissolved in PBS (Sigma) was added to a final concentration of 0.5 mM to each well. plates were incubated for 4 hours at 37°C to then read in a BioTek Synergy HT plate reader (excitation = 560 nm, emission = 590 nm). Mean values derived from parallel triplicate wells were normalized to the mean value obtained for triplicate cell-free media control wells. Mean values for vehicle-treated control wells were assigned 100% viability and cell viability for inhibitor exposed wells calculated accordingly. Survival data were plotted using a three-parameter regression curve fit in GraphPad Prism 8 software.

shRNA expressing constructs and viral infection. Viruses were packaged using HEK 293T cells. Cells were seeded into 10 cm tissue culture dishes for 24-36 hours, then transfected with packaging plasmids psPAX2, pMD2.G and lentiviral backbone construct at a ratio of 2.5:1:5.5 using the ProFection™ mammalian transfection system according to the manufacturer's instructions (Promega). Transfection media was replaced with culture medium after 16 h incubation. Viral supernatants were harvested 24, 48 and 72 h later, filtered through 0.45-micron syringe filters, supplemented with hexadimethrine bromide to yield a final concentration of 4 μg/ ml (polybrene, Sigma-Aldrich) and either used directly or stored at −80°C. The pGIPZ-shRNA constructs targeting human *RB1* were purchased from Horizon and were designated V2LHS 130606 (shRB1-1), V2LHS 130608 (shRB1-2), V2LHS 340824

(shRB1-3) and V3LHS 340825 (shRB1-6) with sequences TAAGTTCACATGTCCTTTC, TTAACTGAAATGAAATCAC, AATCTTGCATCTAGATCTT and TAATTTCTGACATAATGCA respectively. The pGIPZ-shRNA constructs targeting human 53BP1 were designated V2LHS 56191 (sh53BP1-1) and V3LHS 318807 (sh53BP1-2) with sequences TCCTTATTAGTACTCCGTC and TACTTTTCTTTCTACTTCT, respectively. For viral transduction cells were seeded into 6-well plates were incubated with polybrene supplemented viral supernatant in complete media overnight. The next day, media replaced with fresh media. Cells were subjected to puromycin selection 48 hrs post-infection to select for virus uptake.

Flow cytometry assisted cell cycle analysis. Cells were plated in 6-well dishes at a concentration of 1.5 $x10^5$ cells per well and treated in parallel to cells seeded for experiments to document reposes to thymidine treatment. Thymidine (Sigma), dissolved in Phosphate Buffered Saline (PBS), was used at a final concentration of 2 mM. Cells were harvested eight hours later, fixed using 70% ethanol for a minimum of 12 hours. For analysis, cells were treated with 10 μg/ml DNase and protease-free RNAse A for 10 minutes at room temperature, followed by the addition of propidium iodide (Sigma) to a final concentration of 200 μg/ml containing. Samples were analysed using a FACSDiva Fortessa X20 (Becton Dickinson) flow cytometer.

Xenograft models. Breeder pairs of NRG (NOD.Cg-Rag1tm1Mom Il2rgtm1Wjl/Szj) immunodeficient mice were from the Charles River, UK. Procedures were carried out in accordance with the Animals (Scientific Procedures) Act 1986 and according to United Kingdom Coordinating Committee on Cancer Research guidelines for animal experimentation [6], under UK Home Office License, with UCL (University College London)'s Animal Welfare Ethical Review Body (AWERB) approval.

OHSN and CAL72 osteosarcoma-derived cells resuspended at a concentration of 8×10^7 cells/ ml in sterile PBS and 50 μl (4X 10^6 cells) were injected s. c. into the right flanks of host animals. Once tumours reached ~100 mm^3, assessed using digital callipers, mice were randomly assigned to treatment groups and treated once daily p. o. using 0.33 mg/ kg talazoparib or vehicle formulated in 10% Dimethylacetamide, 6% Solutol, and 84% PBS. Treatment was administered for three consecutive weeks, on 5 consecutive days (Monday to Friday) each, in accordance with AWERB guidance limiting the allowable number of doses to a maximum of 15 per animal. Mice were weighed, and tumour dimensions were determined twice weekly using digital callipers. Tumour volume was calculated using (length × $width^2$)/2. Tumour growth was assessed relative to its respective initial size. All experiments were conducted using humane endpoints in accordance with AWERB guidelines. Assessments

were terminated in accordance with AWERB guidelines. Specifically, mice were euthanised when tumour size reached either a maximum mean diameter of 12 mm or a maximum single dimension of 15 mm.

Whole-genome sequencing (WGS) of osteosarcoma cell lines. Whole-genome sequencing was conducted by Macrogen Europe on the Illumina NovaSeq instrument according to the manufacturer's protocol using 150 bp, paired-end libraries with a PCR free workflow. The average coverage of tumours was 30X.

Processing of WGS. WGS processing was performed using the bcbio-nextgen1.2.4 pipeline (https://zenodo.org/record/4686097#.YPn1U5MzYqJ). Reads were aligned to the GRCh38 reference using bwa-mem (https://arxiv.org/abs/1303.3997v2) (parameters :bwa "-M" "-a", mark duplicates = TRUE, recalibrate = TRUE), and samtools [7], and sambamba [8], to sort bam files and mark duplicate reads. SNVs and indels were called using Mutect2 (version GATK4,4.1.9.0) (https://gatk.broadinstitute.org/hc/en-us/articles/360037593851-Mutect2) in tumour mode only (without a matched normal) using the following filters: "panel of normals --gatk4_mutect2_4136_pon.vcf.gz", "--germline-resource af-only-gnomad.hg38.vcf.gz","--af-of-alleles-not-in-resource 0.0000025". Other options used for variant calling included avoiding variants in low complexity regions, polyx stretches, alternate contigs, and repeat regions of high depth. Oxidation and strand artefacts were identified using DKFZ bias filters (https://github.com/DKFZ-ODCF/DKFZBiasFilter). Variants were annotated using Ensembl Variant Effect Predictor [9] (release 102) and converted to the MAF format using vcf2maf (Version 1.6.21) (https://doi.org/10.5281/zenodo.1185418). Only variants with a "PASS" filter and passing the DKFZ filter were retained for signature analysis. Somatic copy-number variants were identified with ASCAT [10]. Lack of germline information for the available cell lines or normal contamination makes it difficult to distinguish loss-of-heterozygosity (LOH) from germline homozygosity**.** Allele counts were modelled as binomially distributed with a homozygous probability of 0.99. SNPs with > 0.3 probability of being heterozygous were re-classified as heterozygous.

Processing of whole-exome sequences (WES). Bam files were accessed from the European Genome-Phenome Archive (https://ega-archive.org) Accession: EGAD00001001039. Sequences were processed using Mutect2 (version GATK4,4.1.9.0) (https://gatk.broadinstitute.org/hc/en-us/articles/360037593851-Mutect2) as described under WGS with the exceptions of using an appropriate panel of normals and filters for exome sequencing. Arguments: "panel of normals – somatic-b37_Mutect2-exome-panel.vcf" "—

germline-resource somatic-b37_af-only-gnomad.raw.sites.vcf","--af-of-alleles-not-in-resource 0.0000025".

Processing of SNP6 microarrays. CEL files were accessed from COSMIC (https://cancer.sanger.ac.uk/cell_lines). LogR and BAF values were generated using PennCNV [11] and Affymetrix Power Tools as described here: https://www.crick.ac.uk/research/labs/peter-van-loo/software. Copy number was initially called using ASCAT [10], with a segmentation penalty of 70. Due to a lack of germline information for the available cell lines, and a lack of normal contamination, loss of heterozygosity cannot be distinguished from germline homozygosity, which leaves segmentation blind-spots without inferred heterozygous SNPs in the tumour. To remedy this, called copy number segments that had a depletion of predicted heterozygous SNPs (<20% SNPs heterozygous) had as many SNPs with B-allele frequency closer to 0.5 re-designated as heterozygous to reach 20% heterozygous SNPs. With these extra heterozygous SNPs, copy number was recalled using ASCAT, recovering LOH segments.

Mutational signature analysis. Counts of the 96 single-base substitution triplet contexts were generated using sigProfilerMatrixGenerator [1]. Each sample was decomposed into exposures of 46 previously published mutational signatures using sigProfilerSingleSample with default settings [1]. Activities of all signatures identified in a sample were normalized in sum to 1.
The R package scarHRD [12] was used to scores 'genomic scars' in the copy number landscape that are often seen in HRD tumours: telomeric allelic imbalance [13], large LOH segments [14] and transitions between large regions of the genome with divergent copy number [15]. ScarHRD was run for all copy number profiles generated in previous steps.

For mutational signature analysis in tumour sample, mutational data, mutational signature activities, and copy number data from a series of 37 previously published whole-genome sequenced osteosarcomas was downloaded [16]. Mutational signature activities were normalized in sum to 1 in each sample. Homozygous deletions, CN {0,0}, and loss-of-heterozygosity events, CN {>0,0}, were called at *RB1* from the associated copy number data. Combinations of mutations/copy number alterations of *RB1* were compared to COSMIC3 activity.

Statistical Analysis. Statistical hypothesis testing was performed using Microsoft Excel or GraphPad Prism. Two-tailed Student's t-test or one-way Analysis of Variance (ANOVA) tests were used for normally distributed data and two-tailed Mann-Whitney nonparametric test

where data deviated from normality. A Kruskal-Wallis test with Sidak's multiple comparisons correction was used for comparison of RAD51 immunofluorescence data. Survival was analysed by Kaplan-Meier plot, and log-rank (Mantel-Cox) test was used to compare data. Differences with $p<0.05$ were considered statistically significant.

SUPPLEMENTARY REFERENCES

1 Alexandrov, L. B. *et al.* The repertoire of mutational signatures in human cancer. *Nature* **578**, 94-101, doi:10.1038/s41586-020-1943-3 (2020).

2 Nanni, P. *et al.* Bone sarcoma patient-derived xenografts are faithful and stable preclinical models for molecular and therapeutic investigations. *Sci Rep* **9**, 12174, doi:10.1038/s41598-019-48634-y (2019).

3 Zhang, C. *et al.* Signalling involving MET and FAK supports cell division independent of the activity of the cell cycle-regulating CDK4/6 kinases. *Oncogene* **38**, 5905-5920, doi:10.1038/s41388-019-0850-2 (2019).

4 Richardson, E. *et al.* Mechanism-based screen establishes signalling framework for DNA damage-associated G1 checkpoint response. *PLoS One* **7**, e31627, doi:10.1371/journal.pone.0031627 (2012).

5 Stockwell, S. R. & Mittnacht, S. High-Content Imaging and RNAi Screens for Investigating Kinase Network Plasticity. *Methods Mol Biol* **1636**, 133-161, doi:10.1007/978-1-4939-7154-1_10 (2017).

6 Workman, P. *et al.* UKCCCR guidelines for the welfare of animals in experimental neoplasia. *Lab Anim* **22**, 195-201, doi:10.1258/002367788780746467 (1988).

7 Li, H. *et al.* The Sequence Alignment/Map format and SAMtools. *Bioinformatics* **25**, 2078-2079, doi:10.1093/bioinformatics/btp352 (2009).

8 Tarasov, A., Vilella, A. J., Cuppen, E., Nijman, I. J. & Prins, P. Sambamba: fast processing of NGS alignment formats. *Bioinformatics* **31**, 2032-2034, doi:10.1093/bioinformatics/btv098 (2015).

9 McLaren, W. *et al.* The Ensembl Variant Effect Predictor. *Genome Biol* **17**, 122, doi:10.1186/s13059-016-0974-4 (2016).

10 Van Loo, P. *et al.* Allele-specific copy number analysis of tumors. *Proc Natl Acad Sci U S A* **107**, 16910-16915, doi:10.1073/pnas.1009843107 (2010).

11 Wang, K. *et al.* PennCNV: an integrated hidden Markov model designed for high-resolution copy number variation detection in whole-genome SNP genotyping data. *Genome Res* **17**, 1665-1674, doi:10.1101/gr.6861907 (2007).

12 Sztupinszki, Z. *et al.* Migrating the SNP array-based homologous recombination deficiency measures to next generation sequencing data of breast cancer. *NPJ Breast Cancer* **4**, 16, doi:10.1038/s41523-018-0066-6 (2018).

13 Birkbak, N. J. *et al.* Telomeric allelic imbalance indicates defective DNA repair and sensitivity to DNA-damaging agents. *Cancer Discov* **2**, 366-375, doi:10.1158/2159-8290.CD-11-0206 (2012).

14 Abkevich, V. *et al.* Patterns of genomic loss of heterozygosity predict homologous recombination repair defects in epithelial ovarian cancer. *Br J Cancer* **107**, 1776-1782, doi:10.1038/bjc.2012.451 (2012).

15 Popova, T. *et al.* Ploidy and large-scale genomic instability consistently identify basal-like breast carcinomas with BRCA1/2 inactivation. *Cancer Res* **72**, 5454-5462, doi:10.1158/0008-5472.CAN-12-1470 (2012).

16 Behjati, S. *et al.* Recurrent mutation of IGF signalling genes and distinct patterns of genomic rearrangement in osteosarcoma. *Nature Communications* **8**, 15936, doi:10.1038/ncomms15936 (2017).
