# Supplementary material for: Therapeutic vulnerability to PARP1,2 inhibition in RB1-mutant osteosarcoma
Source: Nat Commun. 2021 Dec 3;12:7064. doi: 10.1038/s41467-021-27291-8 (PMC8642453; doi:10.1038/s41467-021-27291-8)
Supplement: Supplementary file 3 — Reporting Summary [file 41467_2021_27291_MOESM3_ESM.pdf]

nature portfolio

Corresponding author(s): Sibylle Mittnacht

Last updated by author(s): Oct 29, 2021

Reporting Summary

Nature Portfolio wishes to improve the reproducibility of the work that we publish. This form provides structure for consistency and transparency in reporting. For further information on Nature Portfolio policies, see our Editorial Policies and the Editorial Policy Checklist.

Statistics

For all statistical analyses, confirm that the following items are present in the figure legend, table legend, main text, or Methods section.

n/a	Confirmed	
☐	☒	The exact sample size (*n*) for each experimental group/condition, given as a discrete number and unit of measurement
☐	☒	A statement on whether measurements were taken from distinct samples or whether the same sample was measured repeatedly
☐	☒	The statistical test(s) used AND whether they are one- or two-sided<br>*Only common tests should be described solely by name; describe more complex techniques in the Methods section.*
☐	☒	A description of all covariates tested
☐	☒	A description of any assumptions or corrections, such as tests of normality and adjustment for multiple comparisons
☐	☒	A full description of the statistical parameters including central tendency (e.g. means) or other basic estimates (e.g. regression coefficient) AND variation (e.g. standard deviation) or associated estimates of uncertainty (e.g. confidence intervals)
☐	☒	For null hypothesis testing, the test statistic (e.g. *F*, *t*, *r*) with confidence intervals, effect sizes, degrees of freedom and *P* value noted<br>*Give P values as exact values whenever suitable.*
☒	☐	For Bayesian analysis, information on the choice of priors and Markov chain Monte Carlo settings
☒	☐	For hierarchical and complex designs, identification of the appropriate level for tests and full reporting of outcomes
☐	☒	Estimates of effect sizes (e.g. Cohen's *d*, Pearson's *r*), indicating how they were calculated

Our web collection on statistics for biologists contains articles on many of the points above.

Software and code

Policy information about availability of computer code

Data collection	Time resolved cell death data collected using an IncuCyteZOOM system, (essenbiosciences). Images were obtained by LSM 510 Zeiss confocal laser-scanning microscope (Nikon C2) and Zeiss Axio imager A1 microscope. The imaging of immunoblotting signals was carried out using an Odyssey CLx (LI-COR system) infrared imaging system or chemiluminescent detection (Pierce). Data for cell cycle analysis were collected using a FACSDiva Fortessa X20 (Becton Dickinson) flow cytometer.
Data analysis	Time resolved cell death data analysed by using an IncuCyteZOOM system, (essenbiosciences). Immunofluoresence images were analysed using Imaris Cell Imaging software (Oxford Instruments) and Microsoft Excel. The analysis of immunoblotting signals was carried out using an Odyssey CLx (LI-COR system). Samples for cell cycle were analysed using a FACSDiva Fortessa X20 (Becton Dickinson) flow cytometer. Statistical hypothesis testing was performed using Microsoft Excel or GraphPad Prism 8.<br>WGS processing was performed using the bcbio-nextgen1.2.4 pipeline (https://zenodo.org/record/4686097#.YPn1U5MzYqJ. Reads were aligned using bwa-mem (https://arxiv.org/abs/1303.3997v2) (parameters :bwa "-M" "-a", mark duplicates = TRUE, recalibrate = TRUE), and samtools 7, and sambamba 8 was used to sort bam files and mark duplicate reads. SNVs and indels were called using Mutect2 (version GATK4,4.1.9.0) (https://gatk.broadinstitute.org/hc/en-us/articles/360037593851-Mutect2) in tumour mode only (without a matched normal) using the following filters: "panel of normals --gatk4_mutect2_4136_pon.vcf.gz", "--germline-resource af-only-gnomad.hg38.vcf.gz","--af-of-alleles-not-in-resource 0.0000025". Copy number was called using ASCAT 10, with a segmentation penalty of 70. Mutational signature 96 single-base substitution triplet contexts were generated using sigProfilerMatrixGenerator. The R package scarHRD 12 was used to scores 'genomic scars' in the copy number landscape that are often seen in HRD tumours.

For manuscripts utilizing custom algorithms or software that are central to the research but not yet described in published literature, software must be made available to editors and reviewers. We strongly encourage code deposition in a community repository (e.g. GitHub). See the Nature Portfolio guidelines for submitting code & software for further information.

Data

Policy information about availability of data

All manuscripts must include a data availability statement. This statement should provide the following information, where applicable:

- Accession codes, unique identifiers, or web links for publicly available datasets
- A description of any restrictions on data availability
- For clinical datasets or third party data, please ensure that the statement adheres to our policy

The authors declare that the data support the findings of this study are available within this Article, the Supplementary information or from the authors upon reasonable requests. Source data are provided with this paper. Sequencing data used in this study are available in the European Nucleotide Archive (ENA), hosted by EMBL-EBI (https://www.ebi.ac.uk/ena/browser/home) under study accession code PRJEB47683.

Field-specific reporting

Please select the one below that is the best fit for your research. If you are not sure, read the appropriate sections before making your selection.

[x] Life sciences [ ] Behavioural & social sciences [ ] Ecological, evolutionary & environmental sciences

For a reference copy of the document with all sections, see nature.com/documents/nr-reporting-summary-flat.pdf

Life sciences study design

All studies must disclose on these points even when the disclosure is negative.

Sample size	Sample sizes were indicated in the legend of each Figure and Supplementary Figure. No statistical methods were used to predetermine sample sizes.
Data exclusions	Data were not excluded in the analysis.
Replication	All data types were independently reproduced with independent experiment numbers given in the Figure legends and Figures. Experiments reporting clonogenic survival, real time death assays and all microscopy assessments were undertaken involving intra-experimental duplicates. Day-5 viability assessments were undertaken involving intra-experimental triplicates.
Randomization	All mice were randomly allocated into experimental groups before the start of the treatment. For cell based experiments in vitro seeded cell populations were randomly allocated to treatment groups prior to treatment. Key findings were repeated with independent experiments.
Blinding	For observer- based microscopy data assessment and collection, observers were blinded to sample identities. Blinding was not relevant to the genomic data and automated image analysis because investigator's bias would not affect the data collection. Data analysis was not blinded because it is strictly quantitative.<br>For in vivo tumour size assessment , where analysis relied on observer based measurements, data collection was undertaken in a double blinded fashion , with assessors unaware of treatment groupings or prior determined measurements.

Reporting for specific materials, systems and methods

We require information from authors about some types of materials, experimental systems and methods used in many studies. Here, indicate whether each material, system or method listed is relevant to your study. If you are not sure if a list item applies to your research, read the appropriate section before selecting a response.

Materials & experimental systems

n/a	Involved in the study	
[ ]	[x]	Antibodies
[ ]	[x]	Eukaryotic cell lines
[x]	[ ]	Palaeontology and archaeology
[ ]	[x]	Animals and other organisms
[x]	[ ]	Human research participants
[x]	[ ]	Clinical data
[x]	[ ]	Dual use research of concern

Methods

n/a	Involved in the study	
[x]	[ ]	ChIP-seq
[ ]	[x]	Flow cytometry
[x]	[ ]	MRI-based neuroimaging

Antibodies

Antibodies used	[anti-RB1 (4H1)] WB, IP, IF, IHC, F, ChIP; Cell Signalling Technology; #9309; Mouse monoclonal; dilution 1:2000<br>[anti-phospho-CHK1 (Ser345) (133D3)] WB, F, IF; Cell Signaling Technology; #2348; Rabbit monoclonal; dilution 1:1000<br>[anti-phospho-CHK2 (Thr68) (C13C1)] F; Cell Signaling Technology; #12812; Rabbit monoclonal; dilution 1:1000

anti-CHK1 (2G1D5) WB; Cell Signaling Technology; #2360; Mouse monoclonal; dilution 1:1000
anti-CHK2 (1C12) WB, IHC, IF; Cell Signaling Technology; #3440; Mouse monoclonal; dilution 1:1000
anti-53BP1 (EPR2172) WB, IHC, IF, F; Abcam; #175933; Rabbit monoclonal; dilution 1:1000
anti-GAPDH (6C5) WB, IF; Abcam; #8245; Rabbit monoclonal; dilution 1:10000
anti-RAD51 WB, IP, IF, IHC; Abcam; #176458; Rabbit polyclonal; dilution 1:850
anti-phosphorylated histone-H2AX (Ser139) (JBW301) WB, IP, IF, IHC; Merck Millipore; #05-636; Mouse monoclonal; dilution 1:1000
anti-Vinculin (hVIN-1) WB, IF, IHC; Sigma #V9131; Mouse monoclonal; dilution 1:1000

[anti-Human Alexa Fluor 488-coupled secondary antibodies] IF, F; Life Technologies; A-11013; Goat / IgG
[anti-Human Alexa Fluor 568-coupled secondary antibodies] IF, ICC, IHC; Life Technologies; A-21090; Goat / IgG
Goat Anti-Rabbit IgG IRDye 800CW-conjugated (LI-COR) secondary antibody
Goat Anti-Mouse IgG IRDye 680RD-conjugated (LI-COR) secondary antibody
Goat anti-Mouse IgG HRP-conjugated secondary antibody
Goat anti-Rabbit IgG HRP-conjugated secondary antibody

Validation

All primary antibodies used in this study were validated by the manufacture. Validation data / citations can be found on the manufacture website by searching the antibody catalog number provided in materials and methods section of our manuscript.

[anti-RB1 (4H1)] application: WB, IP, IF, IHC, F, ChIP; species reactivity: Human, Monkey, Bovine, Pig;
https://www.cellsignal.co.uk/products/primary-antibodies/rb-4h1-mouse-mab/9309

[anti-phospho-CHK1 (Ser345) (133D3)] application: WB, F, IF; species reactivity: Human, Mouse, Rat, Monkey;
https://www.cellsignal.co.uk/products/primary-antibodies/phospho-chk1-ser345-133d3-rabbit-mab/2348

[[anti-phospho-CHK2 (Thr68) (C13C1)] application: F; Species Reactivity: Human;
https://www.cellsignal.com/products/antibody-conjugates/phospho-chk2-thr68-c13c1-rabbit-mab-pe-conjugate/12812

anti-CHK1 (2G1D5) application: WB; Species Reactivity:Human, Mouse, Rat, Monkey;
https://www.cellsignal.co.uk/products/primary-antibodies/chk1-2g1d5-mouse-mab/2360

anti-CHK2 (1C12) application: WB, IHC, IF; Species Reactivity:Human, Monkey;
https://www.cellsignal.co.uk/products/primary-antibodies/chk2-1c12-mouse-mab/3440

anti-53BP1 (EPR2172) application: WB, IHC, IF, F; Species Reactivity: Human, Mouse, Rat;
https://www.abcam.com/53bp1-antibody-epr21722-ab175933.html

anti-GAPDH (6C5) application: WB, IF; Species Reactivity: Human, Mouse, Rat;
https://www.abcam.com/gapdh-antibody-6c5-loading-control-ab8245.html

anti-RAD51 application: WB, IP, IF, IHC; Species Reactivity:Mouse, Human, Xenopus laevis, Chinese hamster;
https://www.abcam.com/rad51-antibody-ab176458.html

anti-phosphorylated histone-H2AX (Ser139) (JBW301) application: WB, IP, IF, IHC; Species Reactivity: Human, Mouse, Rat;
https://www.merckmillipore.com/GB/en/product/Anti-phospho-Histone-H2A.X-Ser139-Antibody-clone-JBW301,MM_NF-05-636

anti-Vinculin (hVIN-1) application: WB, IF, IHC; Species Reactivity: Human, Mouse, Rat, Chicken, Frog;
https://www.sigmaaldrich.com/GB/en/product/sigma/v9131?
gclid=EAIaIQobChMIv7jUl9Pv8wIVB57tCh22pwuTEAAYAiAAEgKyX_D_BwE

Eukaryotic cell lines

Policy information about cell lines

Cell line source(s)	OS panel lines, HEK 293, CAPAN1, SUM149: Wellcome Sanger Institute Cell model resource;
Authentication	OS cell line panel, HEK293, CAPAN1, SUM149: STR profiling using the GenePrint 10 system (Promega), PDX models: DNA Fingerprinting (POWERPLEX ESX 17 Fast System, Promega) against the profile of the original PDX
Mycoplasma contamination	OS panel lines, HEK 293, CAPAN1, SUM149: negative in preserved working stocks using PCR based assessment, PDX models: negative in preserved working stock using Mycoalert Mycoplasma Detection Kit, Lonza
Commonly misidentified lines (See ICLAC register)	No commonly misidentified lines were used in this study.

Animals and other organisms

Policy information about studies involving animals; ARRIVE guidelines recommended for reporting animal research

Laboratory animals	Mus musculus, laboratory strain NRG (NOD.Cg-Rag1tm1Mom Il2rgtm1Wjl/Szj), mixed sex, adult (> 8 weeks pp)
Wild animals	Wild animals were not involved in this study.
Field-collected samples	Field-collected samples were not involved in this study.
Ethics oversight	Procedures were carried out in accordance with the Animals (Scientific Procedures) Act 1986 and according to United Kingdom Coordinating Committee on Cancer Research guidelines for animal experimentation 6, under UK Home Office License, with UCL (University College London)'s Institutional Animal Welfare Ethical Review Body (AWERB) approval.

Note that full information on the approval of the study protocol must also be provided in the manuscript.

Flow Cytometry

Plots

Confirm that:

- [x] The axis labels state the marker and fluorochrome used (e.g. CD4-FITC).
- [x] The axis scales are clearly visible. Include numbers along axes only for bottom left plot of group (a 'group' is an analysis of identical markers).
- [x] All plots are contour plots with outliers or pseudocolor plots.
- [x] A numerical value for number of cells or percentage (with statistics) is provided.

Methodology

Sample preparation	whole cell population processed using ethanol fixation, followed removal of Ribonucleic acids using RNAse A and nuclei acid staining using propidium iodide
Instrument	BD FACSDiva Fortessa X20
Software	FACSDiva
Cell population abundance	not relevant, one-dimesional analysis
Gating strategy	none used

- [ ] Tick this box to confirm that a figure exemplifying the gating strategy is provided in the Supplementary Information.
